# Lung shunt fraction calculation using ^99m^Tc-MAA SPECT/CT imaging for ^90^Y microsphere selective internal radiation therapy of liver tumors

**DOI:** 10.1186/s13550-021-00837-z

**Published:** 2021-09-28

**Authors:** Mike F. Georgiou, Russ A. Kuker, Matthew T. Studenski, Preeti P. Ahlman, Megan Witte, Lorraine Portelance

**Affiliations:** 1 Department of Radiology, Miller School of Medicine, University of Miami, 1611 NW 12th Avenue, JMH C-248, Miami, FL 33136 USA; 2 Department of Radiation Oncology, Miller School of Medicine, University of Miami, Miami, FL USA; 3 Department of Biomedical Engineering, University of Miami, Miami, FL USA

**Keywords:** Lung shunt fraction, SIRT, ^99m^Tc-MAA, ^90^Y

## Abstract

**Background:**

^99m^Tc-macroaggregated albumin (^99m^Tc-MAA) scintigraphy is utilized in treatment planning for Yttrium-90 (^90^Y) Selective Internal Radiation Therapy (SIRT) of liver tumors to evaluate hepatopulmonary shunting by calculating the lung shunt fraction (LSF). The purpose of this study was to evaluate if LSF calculation using SPECT/CT instead of planar gamma camera imaging is more accurate and if this can potentially lead to more effective treatment planning of hepatic lesions while avoiding excessive pulmonary irradiation.

**Results:**

LSF calculation was obtained using two different methodologies in 85 cases from consecutive patients intended to receive ^90^Y SIRT. The first method was based on planar gamma camera imaging in the anterior and posterior views with geometric mean calculation of the LSF from regions of interest of the liver and lungs. The second method was based on segmentation of the liver and lungs from SPECT/CT images of the thorax and abdomen. The differences in planar imaging versus SPECT/CT derived LSF values along with the estimated absorbed lung mean dose (LMD) were evaluated. The LSF values were higher in planar imaging versus SPECT/CT in 81/85 cases, with a mean value of 8.5% vs. 4.6% respectively; the difference was statistically significant using a paired t-test (alpha = 0.05). In those patients who received SIRT, the estimated absorbed LMD calculated with planar imaging was significantly higher than with SPECT/CT (t-test, *P* < 0.005). Repeated phantom experiments using an anthropomorphic torso phantom with variable ^99m^Tc activity concentrations for the liver and lungs were performed with the standard patient protocol, demonstrated improved accuracy of the LSF calculation based on SPECT/CT than planar imaging (mean overestimated value of 6% vs. 26%).

**Conclusions:**

This study demonstrates that LSF calculation using planar imaging can be significantly overestimated while calculation using SPECT/CT imaging and appropriate segmentation tools can be more accurate. Minimizing the errors in obtaining the LSF can lead to more effective ^90^Y SIRT treatment planning for hepatic tumors while ensuring the lung dose will not exceed the standard acceptable safety thresholds.

## Background

Selective internal radiation therapy (SIRT) using Yttrium-90 (^90^Y) labeled microspheres has been used widely and successfully for the treatment of patients with primary hepatocellular carcinoma (HCC) or metastatic liver tumors. The process of injecting radiolabeled microspheres in SIRT via the hepatic arterial branches limits undesirable exposure to the surrounding normal liver parenchyma and thereby can potentially deliver higher radiation dose to unresectable liver tumors than an external radiation beam [1]. SIRT has been shown to be safe and effective treatment [2–4] and could lead to high tumor responses and time to progression or progression-free survival in certain patient populations [5]. Contraindications to ^90^Y SIRT include the presence of gastrointestinal shunts that cannot be corrected by catheter embolization techniques [6] and hepatopulmonary shunts that can potentially cause radiation pneumonitis [7, 8]. The dose constraint to the lungs is considered a lung mean dose (LMD) of 30 Gy from a single treatment or cumulative dose of 50 Gy when multiple treatments are delivered [9, 10].

^99m^Tc-macroaggregated albumin (^99m^Tc-MAA) scintigraphy is utilized as a pre-^90^Y SIRT simulation of the microsphere distribution to evaluate gastrointestinal shunts and to estimate quantitatively the hepatopulmonary shunting, which could potentially result in undesired damage to the lungs or other organs. Essentially ^99m^Tc-MAA particles which closely resemble in size the ^90^Y microsphere dimensions are used as a surrogate means to study intrahepatic and extrahepatic deposition of activity [11]. Diagnostic angiography is used in this work-up to identify arterial anatomic variants that can be readily embolized prophylactically to prevent complications from gastrointestinal shunts [12].

Conventionally, hepatopulmonary lung shunt fraction (LSF) has been calculated in ^99m^Tc-MAA planar scintigraphy [11] using a simple geometric mean formulation of counts obtained by defining regions of interest (ROIs) of the liver and lungs in the anterior and posterior views [13]. Planar imaging and thereby 2D quantification suffers from inherent differences in tissue density and attenuation between the lungs and the liver and also inaccurate delineation of overlapping structures [13]. Furthermore, ^99m^Tc-MAA uptake in the dome of the liver may introduce radioactive counts to the base of the lungs and in turn introduce a bias in the LSF calculation [13, 14].

In contrast to planar ^99m^Tc-MAA imaging, SPECT/CT imaging can provide volumetric information with improved 3D visualization that enables accurate definition of the liver, lungs and other structures. Furthermore, the application of CT-based attenuation correction and scatter compensation of the SPECT images can improve spatial resolution, contrast and quantitative accuracy.

A number of studies reported an overall overestimation in 2D LSF from planar imaging as compared to 3D LSF from SPECT/CT [13–16]. These studies also addressed the issues and artifacts that can introduce a bias in the 3D LSF calculations, such as lung mismatch between SPECT and CT and liver activity spillover into the lungs due to breathing. Limitations of these studies include not adequately addressing the importance of the regional distribution of the tumor burden in the liver and its relative impact on the LSF calculation, and, addressing the question of when is the more accurate 3D LSF calculation important in potentially affecting treatment management.

The aim of this study was to retrospectively analyze pre-^90^Y ^99m^Tc-MAA data, acquired with our standard protocol using planar and SPECT/CT imaging, to compare the corresponding LSF calculations and evaluate their significance in accurately obtaining LMD values that could aid the treatment planning of SIRT.

## Materials and methods

### Patients

This was a retrospective study that included 85 ^99m^Tc-MAA pre-^90^Y cases from a total of 83 patients with 2 patients who had the study twice. The determination if a patient should receive ^90^Y SIRT was made by a multidisciplinary team at our institution consisting of experts from medical oncology, radiation oncology, nuclear medicine and interventional radiology. These patients had histologically proven unresectable liver tumors from either primary HCC carcinoma or liver metastases from other cancers. In our study, resin microspheres (SIR-Spheres; Sirtex Medical, Woburn, MA) were used for patients with liver metastases and glass microspheres (TheraSphere; Boston Scientific, Marlborough, MA) for patients with primary HCC. In this cohort of patients, by the time this manuscript was prepared, 75 patients had received SIRT, 31 with SIR-Spheres and 44 with TheraSphere microspheres respectively. One patient had TheraSphere treatment twice. The clinical characteristics of the patient population are described in Table 1. Institutional review board (e-prost 20130430) approval was obtained for conducting this research study.

**Table 1:** Characteristics of the cohort

No. Sex, Age, y	All	Male	Female
Patients (n1) ^99m^Tc-MAA	83	53	30
Mean Age (y)	66.5	67.2	65.4
Mean BMI	27.8	27.7	27.9
Patients (n2) ^90^Y treatment	75	49	26
SIR-Spheres	31	14	17
TheraSphere	44	35	9

### Angiography

The angiographic mapping procedure evaluates the vascular anatomy of the liver and allows for the selection of the correct arterial branch for the microsphere injection. Under fluoroscopy, percutaneously inserted catheters assess blood flow of the hepatic artery. This is very important as variant hepatic arterial anatomy is a common occurrence and has been reported in a large percentage of patients [17]. Selective angiography of the superior mesenteric artery assesses anatomic variants and extrahepatic vessels that may lead to non-target dose deposition in surrounding organs and improper estimation of the LSF; prophylactic embolization is typically performed in these cases [18]. In our institution, an Interventional Radiology (IR) angiography suite equipped with Artis Q (Siemens Healthineers; Erlangen, Germany), cone beam computed tomography (CBCT), is used for the pre-^90^Y mapping. Celiac artery and superior mesenteric artery angiograms are performed to evaluate the arterial anatomy and identify any variant anatomy, if present. Selective catheterization of the right and left hepatic arteries is then performed using a 2.8-French microcatheter system (Progreat; Terumo, Somerset, NJ) to further study the anatomy. Lobar CBCT is performed following which vessel feeding the tumor is selected and a superselective angiogram with CBCT is performed to confirm the tumor supply. Prior to ^99m^Tc-MAA injection, prophylactic embolization of any nearby vessels to the planned delivery site, such as the gastroduodenal artery, or the right gastric artery is performed using coils (Concerto; Medtronic, Minneapolis, MN), (Ruby; Penumbra, Alameda, CA) to prevent reflux. ^99m^Tc-MAA (148 ± 14.8 MBq) is then injected into the branch of the hepatic artery supplying the tumor(s) from a site which mimics as close as possible the site selected for the ^90^Y treatment. In our patient cohort, there were 60 injections through the right hepatic artery, 14 through the left hepatic artery and 11 through both the left and right hepatic arteries as a split dose.

### Phantom study

In order to evaluate the process of calculating the LSF using planar and SPECT/CT imaging and to assess their differences, phantom experiments with the anthropomorphic torso phantom (Data Spectrum, Durham, NC) were performed. This specially designed phantom simulates an average to large human torso including chest, abdomen, a spine insert and fillable compartments for the lungs (left lung 900 mL, right lung 1100 mL), liver (1200 mL) and background area (approx. 9450 mL). Several experiments were performed using variable activity concentrations of ^99m^Tc-pertechnetate for the liver and lungs to simulate clinically realistic LSF for a range of values. The actual patient acquisition protocol of pre-^90^Y ^99m^Tc-MAA for both planar and SPECT/CT imaging of the phantom was utilized. Comparison was performed between the 2D LSF derived from planar imaging versus 3D LSF obtained from the SPECT/CT study with and without CT-based attenuation correction.

### ^99m^Tc-MAA image acquisition and reconstruction

Imaging in planar and SPECT/CT mode was performed following a standard protocol for both the phantom and the patients using a Symbia Intevo (Siemens Healthineers; Erlangen, Germany) SPECT/CT system. Low-energy high-resolution collimators were used and a ^99m^Tc energy window of 15% was selected. Planar imaging of the chest and abdomen to include the liver and lungs was performed in the anterior and posterior views (10-min acquisition, 128 × 128 matrix). Subsequently, SPECT/CT of the thorax was performed with every effort made to include the entire lungs in the 40-cm FOV without truncating any part of the liver. In case the entire lungs and the entire liver could not fit in one FOV, then the area of interest was defined using two imaging fields of view to ensure there was no truncation of the lungs and liver. SPECT imaging was performed using a non-circular orbit, step and shoot mode with 64 steps at 30 s/step in a 128 × 128 matrix followed by a low-dose non-diagnostic CT at 130 kVp, 50 effective mAs and 3.0 mm slice thickness. No special instructions were given to the patient other than to breathe normally and avoid large inspirations or expirations, in order to reduce the possibility of breathing artifacts or mis-registrations between the SPECT and CT images. The SPECT and CT images were reconstructed using manufacturer’s standard clinical software: for SPECT, ordered-subset expectation maximization (Flash 3D) was used with 10 iterations, 8 subsets, and Gaussian pre-filter with 9.0 mm FWHM; CT images were reconstructed with filtered-back projection in a 512 × 512 matrix. The SPECT images were corrected for attenuation, using a CT generated attenuation map, and also for scatter and resolution recovery.

### ^99m^Tc-MAA injection to imaging time delay and extra-hepatic activity uptake

It is in general necessary when performing studies with ^99m^Tc-MAA to use a freshly reconstituted kit within the timing window of the manufacturer’s guidelines to ensure its radiochemical purity at the time of injection [19]. Additionally, when ^99m^Tc-MAA is used for SIRT planning, it has been suggested that improved image quality can be achieved by minimizing the injection to imaging elapsed time [19, 20] in order to reduce the potential effects from MAA biodegradation in-vivo over time, such as extra hepatic uptake in other organs. This, however, is sometimes very difficult to achieve in practice for ^90^Y SIRT studies due to logistical delays and possible complications with the procedure. In order to evaluate the impact of the time delay between injection of ^99m^Tc-MAA and imaging in relation to any possible presence of extra-hepatic activity, the SPECT/CT thoraco-abdominal images of all the studies were evaluated by a Nuclear Medicine Physician using MDStation display and review software (Thinking Systems, Clearwater, FL). Cases demonstrating extra-hepatic uptake caused by free ^99m^Tc-pertechnetate were identified and the possible impact of the time delay on the LSF estimation was also evaluated.

### Quantification

The LSF can be calculated using the following equation:

$${\text{LSF }}\left( {\text{\% }} \right) = \frac{{{\text{Counts}}\,{\text{in}}\,{\text{the}}\,{\text{Lungs}}}}{{\left( {{\text{Counts}}\,{\text{in}}\,{\text{the}}\,{\text{Lungs}}\, + \,{\text{Counts}}\,{\text{in}}\,{\text{the}}\,{\text{Liver}}} \right)}} \times { }100{\text{\% }}$$

The above applies for both planar and SPECT/CT imaging, however, the method for obtaining the counts differs.

Planar imaging quantification: Using a standard nuclear medicine workstation, ROIs were drawn manually by a Nuclear Medicine Technologist around the liver and lungs (left lung and right lung separately) in the anterior and posterior views respectively. To avoid including liver counts in the lungs, which would overestimate the LSF, the mediastinum and heart were excluded from the ROIs and a small gap was maintained at the boundary of the dome of the liver and the base of the lungs. The geometric mean of the counts, which is used in the 2D LSF calculation, was then obtained using the following formula:

$${\text{Geometric}}\,{\text{Mean}}\,{\text{of}}\,{\text{Lung}}\,{\text{or}}\,{\text{Liver}}\,{\text{Counts}}\, = \,\sqrt {({\text{Anterior}}\,{\text{Counts}}\, \times \,{\text{Posterior}}\,{\text{Counts}}} ){ }$$

SPECT/CT quantification: The reconstructed SPECT and CT data were imported into the PLANET (DOSIsoft, Cachan, France) clinical software platform for LSF calculation and dosimetric analysis. Segmentation of the lungs took place on the CT images using an automated algorithm based on Hounsfield Unit (HU) threshold values. The liver VOI was created using the following methodologies: (a) The Radiation Oncologist’s manually defined contours on the diagnostic CT or MR were imported into the study as DICOM RT structures along with their corresponding scan. Following image registration between the diagnostic CT or MR images with the CT of the SPECT/CT, the contours were propagated to define the liver on SPECT/CT. The liver VOI could be evaluated by scrolling through the anatomical CT slices in all three planes and adjusted as needed by shrinking or extending the boundaries. (b) The liver VOI was generated automatically by defining a seed point anywhere inside the liver parenchyma on the CT or the SPECT images of the SPECT/CT; a threshold-based adaptive algorithm would then create the liver contours and volume, while corrections could be applied manually in either the CT or the SPECT images interchangeably. With the liver contouring methods, a Boolean ROI operator was applied to exclude any overlap of the liver into the lung thus removing liver count spillover into the lungs that would otherwise bias the LSF calculation.

### Dosimetry calculations

Estimation of the predicted absorbed dose to the lungs for those patients who received ^90^Y microsphere treatment was calculated using the simplified MIRD formula (21):

$${\text{Lung}}\,{\text{Dose}}\,\left( {{\text{Gy}}} \right) = 49.67{ } \times { }\frac{{{\text{Total}}\,{\text{amount}}\,{\text{of}}\,{\text{injected}}\,{\text{activity }}\left( {{\text{GBq}}} \right)}}{{{\text{Lung}}\,{\text{mass }}\left( {{\text{kg}}} \right)}}{ } \times {\text{LSF}}$$

The lung mass (kg) used for MIRD based calculations, both for 2D and 3D lung dose, was obtained from the volume of the lungs (mL) extracted from contouring the CT of the SPECT/CT using the automated threshold algorithm described above multiplied by the lung density assumed to be 0.3 g/mL [22].

Additionally, the lung dose was generated from the SPECT/CT by utilizing voxel-based dosimetry in PLANET based on a local deposition model (LDM) implementation [23, 24]. An advantage of this method over MIRD is that it uses patient-specific lung mass generated inherently by the software from the mean HU of the segmented lung contours.

### Statistical analysis

Excel software (Microsoft) was used for statistical analysis. Descriptive statistics as well as two-sample paired t-tests with assumed unequal variances were used for comparisons between the planar and SPECT/CT LSF values as well as their respective impact on LMD estimates, at a level of *P* < 0.05, which was considered to be statistically significant. Correlative comparisons for LSF and LMD estimates were performed using the Pearson’s correlation coefficient (R^2^).

## Results

### Phantom study

In the performed phantom experiments it was found that the LSF calculated from planar imaging using the geometric mean method was on average overestimated by 26% (range 23–30%) while using SPECT/CT with CT based attenuation correction it was overestimated by an average of only 6% (range 3–9%). When using SPECT/CT without attenuation correction the average overestimation was approximately 15% (range 13–18%). The LSF results of the phantom experiments are shown in Table 2.

**Table 2:** Phantom experiments: LSF in planar imaging vs. SPECT/CT (with % SD from actual LSF in parenthesis)

Exp	Lung KBq/mL	Liver KBq/mL	Actual LSF	2D LSF		3D LSF AC		3D LSF noAC	
1	0.57	25.78	3.58%	4.40%	(22.9%)	3.88%	(8.4%)	4.05%	(13.1%)
2	2.24	60.03	5.85%	7.60%	(29.9%)	6.35%	(8.5%)	6.82%	(16.6%)
3	5.77	122.10	7.30%	9.30%	(27.4%)	7.78%	(6.6%)	8.24%	(12.9%)
4	7.47	123.30	9.21%	11.50%	(24.9%)	9.48%	(2.9%)	10.42%	(13.3%)
5	8.94	146.15	9.23%	11.70%	(26.8%)	9.68%	(4.9%)	10.39%	(12.6%)
6	11.32	157.40	10.70%	13.40%	(25.2%)	11.45%	(7.0%)	12.57%	(17.5%)

Volumes: L Lung = 900 mL, R Lung = 1100 mL, Liver = 1200 mL

Range of Injected Activity: Lungs: (1.15–22.64) MBq; Liver: (30.93–188.89 MBq)

### Patient studies: LSF calculations and impact on lung dose and treatment planning

**Fig. 1:**
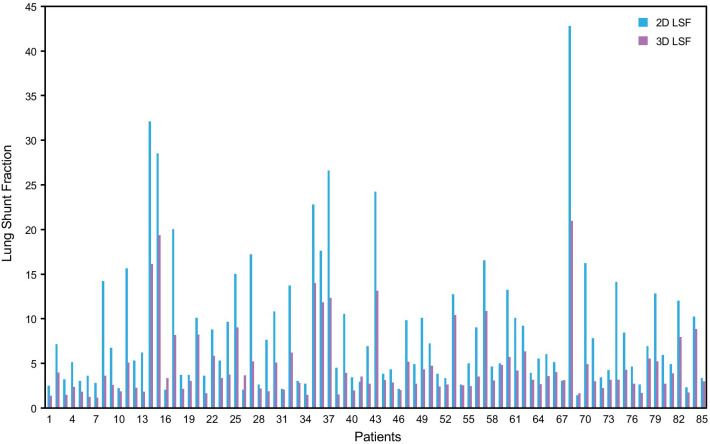
Comparison of ^99m^Tc-MAA planar imaging LSF vs. SPECT/CT derived LSF in 85 cases

**Fig. 2:**
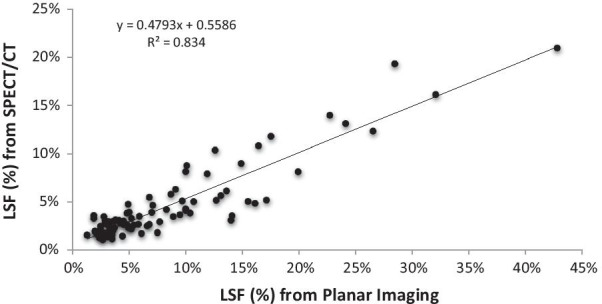
Linear correlation between planar imaging vs. SPECT/CT derived LSF

The mean LSF from planar imaging as compared to SPECT/CT was 8.5% (range 1.4 to 42.8%) and 4.6% (range 1.1 to 20.9%) respectively (Fig. 1). The difference was statistically significant (*P* < 0.001). The 2D LSF was higher than the 3D LSF in 81/85 (95.3%) of the cases with a strong correlation between the two approaches (R^2^ = 0.83; Fig. 2), while the individual percent difference varied by case. In a small sub-group of cases (4/85) with relatively low planar LSF, it was found that SPECT/CT yielded higher LSF, with a non-statistically significant difference (mean LSF 2D = 2.0%, mean LSF 3D = 3.0%). This sub-group presented with a focal right hepatic lobe lesion close to the diaphragm that could potentially bias the 3D LSF calculation from respiratory movement and liver activity spillover inside the lungs.

In our patient cohort, the majority of cases 59/85 (69%) had 2D LSF < 10%, 19/85 (22%) had 2D LSF between 10 and 20%, and 7/85 (8%) cases had 2D LSF > 20%. In all three of these sub-groups there was statistically significant difference between 2D and 3D derived LSF (*P* < 0.001). For the 7/85 cases with 2D LSF > 20% which would be a contraindication in SIR-Spheres treatment planning, even if the calculated LMD would be < 30 Gy, the 3D LSF was < 20% in 6/7 and in only one case the 3D LSF remained > 20% (Patient #68: 2D LSF of 42.8% and 3D LSF of 20.9%).

**Fig. 3:**
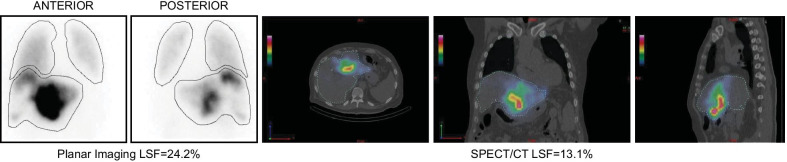
Significantly higher LSF of 24.2% from ^99m^Tc-MAA planar imaging versus LSF of 13.1% from ^99m^Tc-MAA SPECT/CT (patient 43; Table 3)

The importance of % LSF overestimation in planar imaging versus SPECT/CT has to be viewed with clinical perspective and in relation to how it could potentially affect not only the lung dose but also the calculated dose delivered to the tumors and surrounding healthy liver tissue. As an example, Patients #4 and #12 had 2D LSF of 5.1% and 6.2% versus 3D LSF of 2.3% and 1.8% respectively representing > 100% overestimation when in fact the difference in absolute value is very small and would only minimally affect the intended treatment plan whether be ^90^Y resin or glass microspheres. In other cases, it is observed that both percent LSF difference and actual difference in value is significant enough to potentially affect lung dose calculations and dosimetric calculations for the treatment dose of the liver tumors. Examples include Patient #11 with 2D LSF of 15.6% and 3D LSF of 5.1%, Patient #27 with 2D LSF of 17.2% and corresponding 3D LSF of only 5.2%, and Patient #32 with 2D LSF of 13.7% and corresponding 3D LSF of 6.2%. Some patients with 2D LSF > 20% received ^90^Y SIRT with a modulated prescribe dose that could have been potentially better optimized had the more accurate 3D LSF been available at the time of treatment; examples include patients #17 (resin), #37 (resin) and #43 (glass) with 2D LSF 20.0%, 26.6%, 24.6% and corresponding 3D LSF 8.2%, 12.3% and 13.1% respectively (Fig. 3 shows Patient #43).

**Fig. 4:**
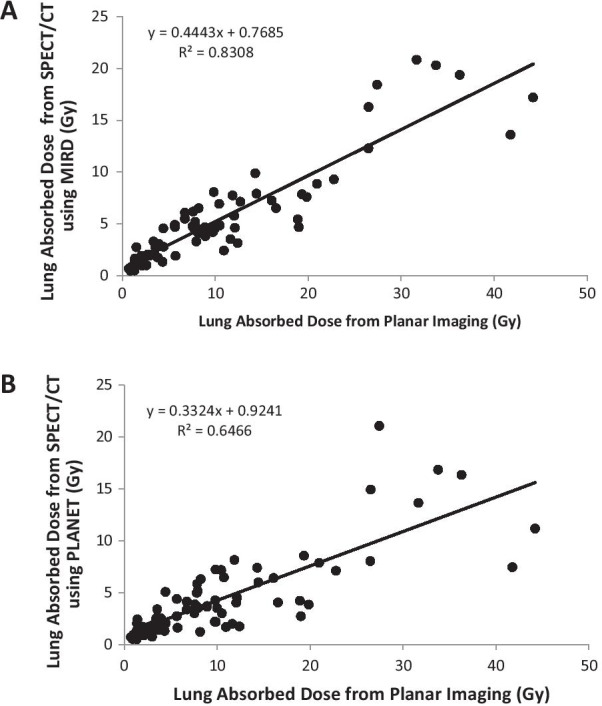
**A** Linear correlation of lung absorbed dose from planar imaging vs. SPECT/CT (MIRD formulation). **B** Linear correlation of lung absorbed dose from planar imaging vs. SPECT/CT (PLANET software)

In this patient cohort, 75 patients received SIRT with 31 SIR-Spheres and 44 TheraSphere. The mean lung mass in this patient population was 0.74 kg (range 0.32 to 1.70 kg). The ^90^Y treatment administered activities ranged from 0.28 to 5.32 GBq with a mean value of 1.81 GBq. The mean LMD as calculated from 2D LSF was 10.48 Gy (range 0.72–44.2 Gy) while for 3D LSF it was 5.43 Gy (range 0.48–20.82 Gy) using MIRD calculations and 4.40 Gy (range 0.5–21.1 Gy) using the PLANET LDM implementation respectively. The difference between 2D LMD calculations and either 3D MIRD or 3D LDM was statistically significant using two-tail t-test with unequal variances (*P* < 0.005). The 2D LSF method would result in a calculated LMD > 30 Gy in 5 patients with 3 additional patients having LMD > 25 Gy, for a total of 8/75 (11%) which would be concerning or contraindicated for treatment planning. Using the 3D LSF methodology and lung dose calculations based on either MIRD or PLANET LDM, the LMD in all cases was < 21 Gy. The LMD derived from planar imaging and the corresponding LMD from SPECT/CT imaging for the 75 patients who received treatment are plotted in Fig. 4A, B. As expected, the correlation between 2D MIRD and 3D MIRD is higher than the correlation between 2D MIRD and 3D PLANET LDM.

Table 3 shows the referenced patient cases with their corresponding 2D versus 3D LSF values and LMD estimates.

**Table 3:** Treatment planning and differences in lung dose estimates as determined from planar imaging vs. SPECT/CT (LDM) ^99m^Tc-MAA imaging in selected patient cases

Patient No	Planar LSF (%)	SPECT/CT LSF (%)	^90^Y microsphere	Injected Activity (GBq)	Planar LMD (Gy)	SPECT/CT LMD (Gy)
4	5.1	2.3	TheraSphere	1.985	10.00	3.50
11	15.6	5.1	TheraSphere	2.413	41.76	7.46
13	6.2	1.8	SIR-Spheres	2.595	18.90	4.23
14	33.7	16.1	–	–	–	–
15	28.5	19.3	–	–	–	–
17	20.0	8.2	SIR-Spheres	1.092	22.77	7.11
27	17.2	5.2	TheraSphere	0.494	4.35	1.32
32	13.7	6.2	TheraSphere	2.198	16.08	6.42
37	26.6	12.3	SIR-Spheres	0.997	26.47	8.04
43	24.2	13.1	TheraSphere	2.679	36.30	16.37
68	42.8	20.9	–	–	–	–

### ^99m^Tc-MAA injection to imaging time delay and extra-hepatic activity uptake

Even though every effort was made to initiate imaging soon after the ^99m^Tc-MAA injection, this was hindered by practical considerations related to the procedure including the transport of the patients from the IR suite to the NM imaging department. In our cohort of 85 patient cases, the mean time-interval from injection to scan was 100 min with a median of 75 min and a wide range from 20 to 300 min (SD = 70 min). The majority of patients (59%) were imaged in less time than the mean value of 100 min and the rest of the patients within 2 SD from the mean with only 1 patient as an outlier at 300 min. There was no extra-hepatic activity uptake in 53 patients (62.4%); this group included patients imaged from 20 to 180 min post-injection. There were 29 patients (34.1%) with activity in both kidneys; these patients were imaged in a time interval of 30 to 300 min post-injection.

## Discussion

The methodology described in the current study for LSF quantification using pre-^90^Y ^99m^Tc-MAA SPECT/CT imaging provided reliable results in a cohort of patients who underwent work-up prior to receiving ^90^Y SIRT. This study demonstrated that the conventional method for LSF calculation based on 2D planar imaging was generally inaccurate and provided overestimated values. These findings were supported by validation experiments using an anthropomorphic torso phantom scanned under clinically realistic scenarios, including simulating attenuation effects and their impact on LSF calculations. Overall, SPECT/CT imaging of the phantom yielded improved accuracy in LSF values (< 10% above actual value) while planar imaging resulted in overestimations (mean of 26% above actual value). The phantom analysis showed that the SPECT/CT methodology was more accurate with CT attenuation correction of the SPECT images. The analysis performed in clinical studies demonstrated that the 2D LSF was generally overestimated, though by a variable degree depending on the specific characteristics of each case, such as size and location of the hepatic lesions, anatomic morphology of each patient and corresponding attenuation effects and possible artifacts from respiratory motion. Technical reasons that could explain why 2D LSF calculation overestimates the LSF may include the difficulty for the operator to accurately delineate the lungs in planar imaging, either anterior or posterior, and to avoid possible overlap with the liver. The overall statistically significant overestimation of LSF from planar ^99m^Tc-MAA imaging is consistent with the findings of previous studies [13, 15].

The small sub-group of patient cases (4/85) with 3D LSF that was higher than the 2D LSF, demonstrates the possibility that the LSF may not be always overestimated in planar imaging. All four patients presented a focal hepatic lesion near the diaphragm and it is possible that depending on the degree of respiratory movement some liver activity was included in the VOI of the right lung. This finding was persistent even after removing 10 mm of the right lung VOI at the basal boundary. This is the first study to our knowledge to report possible overestimation using SPECT/CT versus planar imaging, while only one study [16] reported small, but yet statistically significant, difference. In all four cases, the 2D LSF values were < 3% with a mean difference from 3D LSF of approximately 1%, which was not statistically significant.

Clinical importance for accurate calculation for LSF from the pre-treatment ^99m^Tc-MAA scan lies in its role for potentially determining a < 30 Gy radiation exposure to the lungs and hence preventing radiation pneumonitis [6]. In this study we performed dosimetric analysis to calculate the predicted LMD from the SPECT/CT images using specialized software (PLANET) for those patients who received SIRT. The calculated LMD based on the ^99m^Tc-MAA SPECT/CT methodology introduced in this study was found to be statistically different from the LMD calculated using the conventional two-dimensional approach. While this may not have impacted the large majority of patients with relatively low LSF values, it could potentially change the prescribed treatment dose in those patients with higher LSF values to a higher treatment dose that could consequently be more effective. As well, in those patients with high enough LSF in planar imaging that did not undergo treatment planning out of concern for causing lung toxicity, the methodology introduced in this study based on SPECT/CT-derived LSF would provide additional options for treatment by possibly prescribing a dose that is appropriately adjusted to ensure it is safe. Perhaps, the outcomes of our method which calculates more accurately the LSF using SPECT/CT that includes the entire lungs and can also perform voxel-based dosimetry with organ specific masses for determination of the treatment dose in the same context, can be applied in the direction of establishing updated upper limit safe standards against pulmonary pneumonitis instead of the currently applied ones [8, 9].

Bias in the LSF calculation using SPECT/CT can be introduced from spillover and displacement of liver counts into the right lung due to respiratory motion. This issue can be further exacerbated when there are liver lesions in the area close to the diaphragm and therefore high uptake of ^99m^Tc-MAA activity. To compensate for this problem, previous investigators employed a number of approaches such as excluding a standard size area from the base of the lung [15, 16], considering only the left lobe of the lung for the calculations and excluding the right lung to avoid the area of spillover [14], and instructing patients to use shallow breathing to limit the amount of mis-registration between CT and SPECT for the attenuation-corrected images and thereby possible spillover [13]. In our methodology, the standardized approach was to perform Boolean exclusion of the liver VOI from the lung VOI so that the lungs would not include spillover activity from the liver. We felt that this approach made the workflow reproducible and not operator dependent; visual inspection in the transverse, sagittal and coronal planes with appropriate triangulation ensured that liver activity was excluded from the lungs.

One of the reasons for performing the pre-^90^Y ^99m^Tc-MAA SPECT/CT study is to detect displaced activity outside of the liver that might potentially be a contraindication for performing ^90^Y SIRT depending on the amount of shunt and potential dose to other organs; commonly uptake of activity in the renal parenchyma can be visualized in the presence of extra hepatopulmonary shunts [13]. Another possible reason for visualizing extra-hepatic activity is ^99m^Tc-MAA breakdown by imaging in prolonged timeframes that exceed the recommended 60-min that lung perfusion protocols commonly recommend. The instability of ^99m^Tc-MAA as time post-injection progresses may in turn lead to an overestimation of the LSF [20] particularly in planar imaging because of the influence from overlapping structures in the body outside of the lungs and liver that might be uptaking ^99m^Tc-MAA activity. A group of investigators suggested the use of perchlorate blockade to prevent stomach uptake of ^99m^Tc-MAA that may be considered as gastric shunting [25]. In our study, every effort possible was made to transport the patients from IR to Nuclear Medicine as early as possible following the intervention in order to avoid possible ^99m^Tc-MAA disintegration due to time delay. This, however, is sometimes difficult due to practical issues related to the patient’s condition and efficiency of the workflow. In our study, the large majority of patients did not demonstrate activity outside of the liver and lungs, while a significant number of patients showed activity in the kidneys. Uptake of ^99m^Tc-MAA in other areas included stomach and thyroid most likely attributable to free pertechnatate while in very few cases there was uptake in the gallbladder, common bile duct and spleen. There was no correlation found between ^99m^Tc-MAA uptake outside the liver or lungs and the time delay from administration of ^99m^Tc-MAA to imaging; a similar finding was previously reported [15].

There is increased evidence based on accumulated experience in SIRT, including methodological improvements, that based on current guidelines the pre-treatment LSF as a single factor in determining the patient’s candidacy for treatment is not sufficient. In some cases, investigators chose to treat patients who had high LSF with the systemic drug Sorafenib [26], a protein kinase inhibitor, and over time the repeated LSF was significantly reduced. Furthermore, pre-treatment LSF alone may not be necessarily predictive of the presence of significant hepatopulmonary shunt post-treatment, as seen in the ^90^Y Bremsstrahlung images, with the possibility of radiation pneumonitis [27].

Our study has some limitations that need to be considered. The phantom experiments confirmed the finding that 2D LSF is overestimated as compared to 3D LSF which is more accurate; however, while the impact of attenuation on LSF calculations was sufficiently demonstrated, it would require a respiratory gating system to simulate breathing artifacts and study the effects of liver activity into the lungs. The dosimetric analysis of patient data and predictive dose calculations for GTV, liver, healthy liver and lungs were performed retrospectively with the objective of validating the SPECT/CT methodology. Since the current practice using planar MAA calculations is based on mostly arbitrary thresholds for lung toxicity, the described SPECT/CT methodology would have to be applied prospectively to evaluate its clinical utility in planning more effective ^90^Y SIRT treatments with greater delivered doses while maintaining reliable pulmonary safety.

## Conclusions

This study demonstrated that ^99m^Tc-MAA SPECT/CT imaging provides a significantly more accurate calculation of LSF than conventional planar gamma camera imaging in patients who will receive ^90^Y SIRT. This finding was validated with phantom experiments which further exhibited that CT based attenuation correction in SPECT/CT contributes to the improved accuracy of LSF. This conclusion can be particularly important in those patients with relatively high LSF who cannot receive SIRT and/or would require dose modulation to prevent pulmonary toxicity. More accurate LSF estimation using SPECT/CT can potentially lead to higher and more effective treatment dose in ^90^Y SIRT while safeguarding against radiation induced lung toxicity.

## Data Availability

The datasets generated and/or analyzed for the current study are available from the corresponding author on reasonable request.

## Abbreviations

MAA: Macroaggregated albumin
^99m^Tc: Technetium-99m
^90^Y: Yttrium-90
SIRT: Selective internal radiation therapy
LSF: Lung shunt fraction
LMD: Lung mean dose
HCC: Hepatocellular carcinoma
ROIs: Regions of interest
IR: Interventional radiology
CBCT: Cone beam computed tomography
AC: Attenuation correction
SPECT: Single photon emission computed tomography
CT: Computed tomography
FWHM: Full-width half-max
LDM: Local deposition model
2D: Two-dimensional
3D: Three-dimensional
SD: Standard deviation
NM: Nuclear medicine
FOV: Field of view
